# Revisiting the impact of public spaces on the mental health of rural migrants in Wuhan: an integrated multi-source data analysis

**DOI:** 10.1186/s12942-024-00365-8

**Published:** 2024-03-07

**Authors:** Feifan Gao, Hanbei Cheng, Zhigang Li, Le Yu

**Affiliations:** 1 School of Public Administration and Policy, Renmin University of China, Beijing, 100872 China; 2 School of Public Policy and Management, Tsinghua University, Beijing, 100084 China; 3 School of Urban Design, Wuhan University, Wuhan, 430072 China; 4 Hubei Provincial Research Centre of Human Settlement Engineering and Technology, Wuhan University, Wuhan, 430072 China

**Keywords:** Public space, Rural migrants, Mental health, Perceived integration, Wuhan

## Abstract

Current research on public spaces and mental health often focuses on the independent relationship of one or more social mediators, neglecting the nuanced implications and serial mechanisms inherent in the progressive social process. Using Wuhan city, China, as a study case with multi-source data, this research applies Multilevel Generalized Structural Equation Modeling and deep learning techniques to explore the differential effects of public spaces with varying degrees of publicness (i.e., typical, semi-, and privately owned) on rural migrants’ mental health. Crucially, this study scrutinizes both explicit (social interaction) and implicit (perceived integration) social mechanisms to revisit the relationships. The findings reveal that not all public spaces equally influence mental health, with typical and privately owned public spaces conferring profound benefits. Notably, public spaces impact mental health chiefly through perceived integration instead of through direct social interaction. Social interaction improves mental health primarily by enhancing perceived integration, suggesting that meaningful connections beyond superficial encounters are critical. In particular, we observed significant social effects in typical and privately owned public spaces but limited social functionality in semi-public spaces. This evidence contributes to the knowledge required to create supportive social environments within public spaces, integral to nurturing inclusive urban development.

**Supplementary Information:**

The online version contains supplementary material available at 10.1186/s12942-024-00365-8.

## Introduction

In recent years, mental problems have emerged as a major public health issue in urban China [1]. Nearly 376 million internal migrants, 26.62% of the population, left their hometowns to live and work in cities [2], experiencing severe mental illness due to their disadvantaged socioeconomic status and unfavorable living conditions [3, 4]. Moreover, frequent mobility often leads to superficial interpersonal relationships, disrupted social ties, and weakened local attachment. Rural migrants, in particular, face difficult situations due to prejudice, discrimination, and marginalization in the host cities, which increases their mental health risks [5]. An increasing number of studies have revealed the psychological advantages of public spaces in reducing anxiety, depression, and distress among urban residents [6, 7]. These studies highlight the importance of social mechanisms, such as social cohesion, place attachment, and sense of community, in promoting mental well-being [8]. However, the evidence regarding the social implications of different types of public spaces and their influence on the mental health of rural migrants in urban China is still limited and even less is known about the nuanced progressive social process (the interplay between explicit and implicit social mechanisms) under the associations above.

In this paper, we aim to contribute threefold to the work on public spaces and the mental health of rural migrants. Firstly, grounded in a traditionally dualistic understanding of public spaces, most research has focused on exploring the health effects of typical public spaces, such as squares, parks, sidewalks, markets, and squares [9], as well as privately owned public spaces, including courtyard gardens and front and backyard activity spaces around residential buildings [3, 10]. However, semi-public spaces, which require an economic threshold for entry, have received less attention. The evidence differentiating the health effects of the three types of public spaces remains insufficient. In contrast to native residents, rural migrants often face both economic (limited income) and social (non-native *hukou* status) barriers that may restrict their access to services and resources, including public spaces [11]. The degree of publicness of these spaces can affect their accessibility, which may result in varied mental health outcomes among rural migrants [12]. Crucially, the potential for encounters in different spaces may contribute to the variance in social benefits, which could be a critical underlying factor for the diverse mental health outcomes described above [13]. Therefore, it is imperative to examine the social and mental effects resulting from various types of public spaces on rural migrants in urban China.

Secondly, the current literature on healthy and inclusive cities overemphasizes the positive impact of public spaces in promoting social interaction while overlooking that such interactions may be fleeting and superficial [13]. Additionally, excessive social interaction can result in social withdrawal or public participation in slavery, obedience, and reluctance, as well as reciprocal feedback burdens, which are not conducive to deep-seated integration and thus have limited effect on mental health benefits [5]. Indeed, social exchange theorists have highlighted the dual nature of social interaction [14]. Recent studies have also found that the social mechanisms behind neighborhood health effects are no longer the independent action of a single social factor but a mixture of multiple social aspects, exhibiting a sequential chain of interlocking links [15]. This phenomenon has proven to be more intricate than previously understood. Despite extensive research on the role of a range of social mediators, including neighborly interactions and perceived integration, there remains a lack of in-depth exploration into the profound interplay between explicit social behaviors (i.e., interactions) and implicit social benefits (i.e., integration) [16]. This idea leaves a theoretical puzzle in its wake. Therefore, it is necessary to deconstruct the intricate social mechanisms and thoroughly investigate how public spaces affect the mental health of rural migrants. This can be achieved by examining the respective roles of these two social factors, with particular attention to uncovering their potential serial social mechanisms in public spaces, to elucidate the potential progressive social processes related to the mental health of rural migrants.

Thirdly, technical limitations constitute another significant reason for the gaps in the existing literature. Existing studies have widely combined geographic data or survey data to analyze the environmental characteristics of typical and semi-public spaces. Contrastly, the evaluation of privately owned public spaces is predominantly based on traditional manual audits and field surveys. Nonetheless, the elements involved in this process are nuanced, covering the building’s external condition (e.g., walls, doors, windows, and stairs) [17] and the surrounding environmental features (e.g., recreational facilities, green areas, sanitation, and spatial layout) [18]. Therefore, the effectiveness of traditional methods for evaluating privately owned public spaces has been questioned due to their high cost, time-consuming nature, limitations in the amount, and potential evaluated bias of the data collected. In the technology-driven era, integrating deep learning algorithms to handle massive image and text data to conduct urban research is becoming an emerging trend. For example, street view images are commonly used to compute green visibility [19], assess streetscape quality [20], and detect urban poverty [21]. Citizen message board data with geolocation tags is also employed to identify neighborhood social governance issues and residents’ emotions [22]. Consequently, considering the research purpose of this paper, we employ deep learning methods and integrate multi-source data (including the social survey data, real estate website imagery, city message board data, and Points of Interest (POIs)) to evaluate public spaces in rural migrants’ residential areas nuancedly. The methodological innovation is another fundamental contribution of this work.

In light of the recent discussion on the social function of public spaces, this study utilizes Multilevel Generalized Structural Equation Modeling, with Wuhan as a case study, to explore the impact of three types of public spaces on the mental health of rural migrants and unravel the underlying social mechanisms. The research extends its scope beyond the examination of typical and privately owned public spaces by including semi-public spaces with specific economic entry requirements, thus closely scrutinizing the ‘liminality’ of public spaces. Moreover, in contrast to the broad discourse on social mechanisms in existing literature, our study focuses on the progressive social process by analyzing the impact of public spaces on explicit social interactions, subsequently influencing the implicit perceived integration, ultimately affecting the mental health of rural migrants. We advocate for nuanced planning and development of public spaces, tailored to their unique social influences, to more effectively address rural migrants’ needs for integration and well-being.

## Literature review

### Refining public space typologies: a spectrum of mental health impacts

The associations between public spaces and mental health have been well-documented. Scholars have reached a consensus that well-established public spaces in neighborhoods featuring highly accessible or high-quality sidewalks [23], parks [24], public transport [25], restaurants [26], and leisure facilities [27] contribute to positive mental health outcomes. According to the degree of publicness, public spaces can be classified as typical, semi-, and privately owned [28]. *Typical public spaces* are openly accessible to everyone, including streets, parks, and public transport [29]. *Semi-public spaces* are commonly consumed areas, including but not limited to bars, clubs, and restaurants [10]. These facilities often target specific demographic groups depending on their interest preferences or socioeconomic status. *Privately owned public spaces* include home courtyards, terraces, gardens, small corners in front of and behind the house, and sidewalks in residential areas with restricted access for outsiders, creating a gated environment [30]. These spatial typologies highlight space’s ‘liminality’ nature and the spatial challenges faced by economically or identity-based disadvantaged groups, such as rural migrants. These challenges include inequality, exclusion, segregation, and monopoly [31], resulting in varied access to geographical opportunities and culminating spatial injustice among migrant groups, thereby exacerbating their mental health disparities. Under the national strategic goal of optimizing the overall health of all citizens, it is imperative to give in-depth attention to this issue. Therefore, it is necessary to investigate the impact of different public space types on the mental health of rural migrants. Upon reviewing the existing literature, we propose the following hypothesis:

**H1:** Public spaces, categorized as typical, semi-, and privately owned, exert significant impacts on the mental health of rural migrants, with different effects depending on their type.

### The interplay of explicit and implicit social mechanisms

The nexus between public spaces and mental health goes beyond a simple correlation. Numerous nuanced pathways contribute to this relationship, including physical activity, residential satisfaction, stress reduction, and spiritual restoration [9]. Notably, social processes have received considerable attention in recent years as a critical mechanism, reflecting the social capacity-building function of public spaces [32]. The ‘*Third Place*’ theory provides a crucial lens for interpreting the relationship [33]. It emphasizes the social benefits of third places (public spaces) apart from primary places (homes) and secondary places (workplaces), arguing that third places (1) facilitate social interactions and neighborly relations, (2) provide a sense of belonging and unity and make people feel anchored in their community, and (3) involvement in third places conduces to positive mental status.

A series of social effects arising from the aforementioned social processes, including social capital, social cohesion, and sense of place, are imperative for an individual’s mental well-being [9]. Among these factors, social interaction and perceived integration are particularly important to the mental health of rural migrants [34]. Being culturally and environmentally marginalized in the host cities, migrants need to establish new networks through social interaction and reconstruct their identity and sense of belonging through perceived integration [31]. Social interaction provides essential support for them by improving intercultural communication, while perceived integration diminishes the isolation among outsiders and enhances their involvement in community activities [35]. These two social elements, fundamental to other social and mental outcomes, play a crucial role in determining migrants’ ability to adjust to their new surroundings.

Specifically, *social interaction* refers to an individual’s explicit social behavior, measured by the frequency of greeting, chatting, playing, eating, and reciprocating with others [36, 37]. Research suggests that increased public access encourages social engagement and face-to-face encounters [38]. Regular interaction with neighbors enhances self-efficacy in coping with mental health problems [39, 40]. Another essential aspect to consider is *perceived integration*, which refers to an individual’s implicit perception of how well migrants get along with their neighbors. It is generally measured by social trust, emotional connection, and discrimination [10, 41, 42]. For instance, street greenery has been shown to improve mental health by strengthening place attachment [16]. Local restaurants provide unique sites for acquiring emotional support and are advantageous for preserving mental well-being [26].

Furthermore, it should be recognized that public spaces with varying spatial attributes, such as publicness, accessibility, safety, comfort, and social diversity, may construct social capacities differently, leading to inconsistent mental health outcomes [13]. According to the ‘*Spaces of Encounter*’ theory, the publicness of spaces determines their accessibility and use by different groups [29]. For instance, typical public spaces are intentionally designed to encourage social interaction across a broad spectrum of individuals, promoting inclusivity and diversity. In contrast, semi- and privately owned public spaces often aim to serve particular demographics, leading to a lack of diversity in terms of ethnic/migrant status, potentially resulting in social exclusion among minorities and impeding their integration [13]. Furthermore, people may feel more comfortable and included in open spaces with less surveillance and management than in private spaces with relatively tight control [28]. To summarize, encounters in typical, semi-, and privately owned public spaces can facilitate intergroup interaction and integration to varying degrees [43], which are widely regarded as the critical social determinants of health [44]. Therefore, the following hypotheses are proposed:

**H2:** Social factors critically mediate the relationship between public spaces and rural migrants’ mental health.

**H2a:** Social interaction specifically serves as an explicit mediating mechanism and varies across three types of public spaces.

**H2b:** The influence of perceived integration, as an implicit mediating mechanism, on mental health also varies among different types of public spaces.

It should be noted that while prior empirical investigation acknowledges that well-established public spaces promote intergroup encounters differently [43], it is still controversial whether mere interaction inevitably brings psychological benefits. On the one hand, previous studies have posited the ‘potentially naïve assumption’ that intergroup contact invariably results in inclusive attitudes of urban locals towards migrants [29]. However, contemporary studies have challenged this viewpoint, contending that in certain instances, locals and migrants may coexist ephemerally in the same spaces without engaging in meaningful interactions [45]. Additionally, migrants may experience prejudice, discrimination, and oppression in interactive environments, which can negatively impact their mental health [5].

On the other hand, social interactions can occasionally be disconcerting or negative. As intergroup interactions intensify, rural migrants perceive increasing social and economic disparities with natives in social comparisons, which upsets their psychological equilibrium [46]. Likewise, social interaction may have negative externalities that increase mental health risks, such as unhealthy behavior contagions, like smoking and overdrinking, peer pressure, and excessive rule-based control [47].

These pieces of evidence suggest that the favorable health outcomes of social interaction depend on its ability to yield positive social benefits, particularly through achieving perceived integration. Furthermore, in examining public spaces and the mental health of rural migrants, both explicit (interaction) and implicit (integration) mechanisms operate not only independently but also in interconnected serial dynamics. Dai and He’s study in Shenzhen investigated the influence of neighborhood social environment (social mix) on subjective well-being, verifying a serial mechanism akin to domino effects between social interaction and social capital [15]. However, it is unclear whether this mechanism is applicable in public spaces. Therefore, we propose the following hypothesis:

**H2c:** In the relationship between public spaces and rural migrants’ mental health, social interaction can also affect mental health through perceived integration, showing a serial mediation mechanism.

### Research in urban China

Under the guidance of the ‘Healthy China’ strategy, urban public spaces are increasingly seen as an essential component of urban infrastructure to enhance the quality of life and promote social harmony [24]. For rural migrants, public spaces serve as a platform for interacting with urban locals, beyond providing necessary daily services [10]. The social significance of public spaces crucially enhances social adaptation and mental well-being among rural migrants [5].

Rural migrants in urban China without official urban *hukou* [11] face social and health inequalities due to spatial injustice [12]. In particular, rural migrants residing in deprived neighborhoods have limited access to high-quality typical public spaces, such as parks and greenways [48], which is linked to poorer mental health compared to urban locals [7]. Additionally, rural migrants have challenges in accessing semi-public spaces, such as pubs and upscale restaurants, potentially causing psychosocial stress [34, 49]. Although privately owned public spaces like courtyards are highly accessible [10], they frequently suffer from poor conditions in disadvantaged neighborhoods, including urban villages and old-dilapidated communities [4]. This paradox of high accessibility coupled with low quality may further deteriorate mental health. For instance, Liu et al.’s research in Guangzhou failed to support the influence of neighborhood cleanliness and public space provision on migrants’ subjective well-being [3]. Recently, the social pathways of public spaces affecting migrants’ mental health have also been widely discussed. The social construction of public spaces, such as green spaces, parks, sidewalks, and squares, has also been advocated [43]. A recent study in Shenzhen demonstrated that migrants’ perceptions of green space significantly and indirectly influenced their mental health by enhancing social cohesion [34].

Despite considerable evidence for the positive impact of typical public spaces on the mental health of rural migrants, the influence of semi- and privately owned public spaces, as well as the differences among these types, is not well-understood. Moreover, current research often consolidates social factors into a single mediator, neglecting the nuanced effects and sequential mechanisms within the social process. Further research is necessary to refine the operationalized forms of social mediators and unpack the linkage from explicit behavior to implicit perception underlying the associations between public spaces and mental health among rural migrants.

**Fig. 1:**
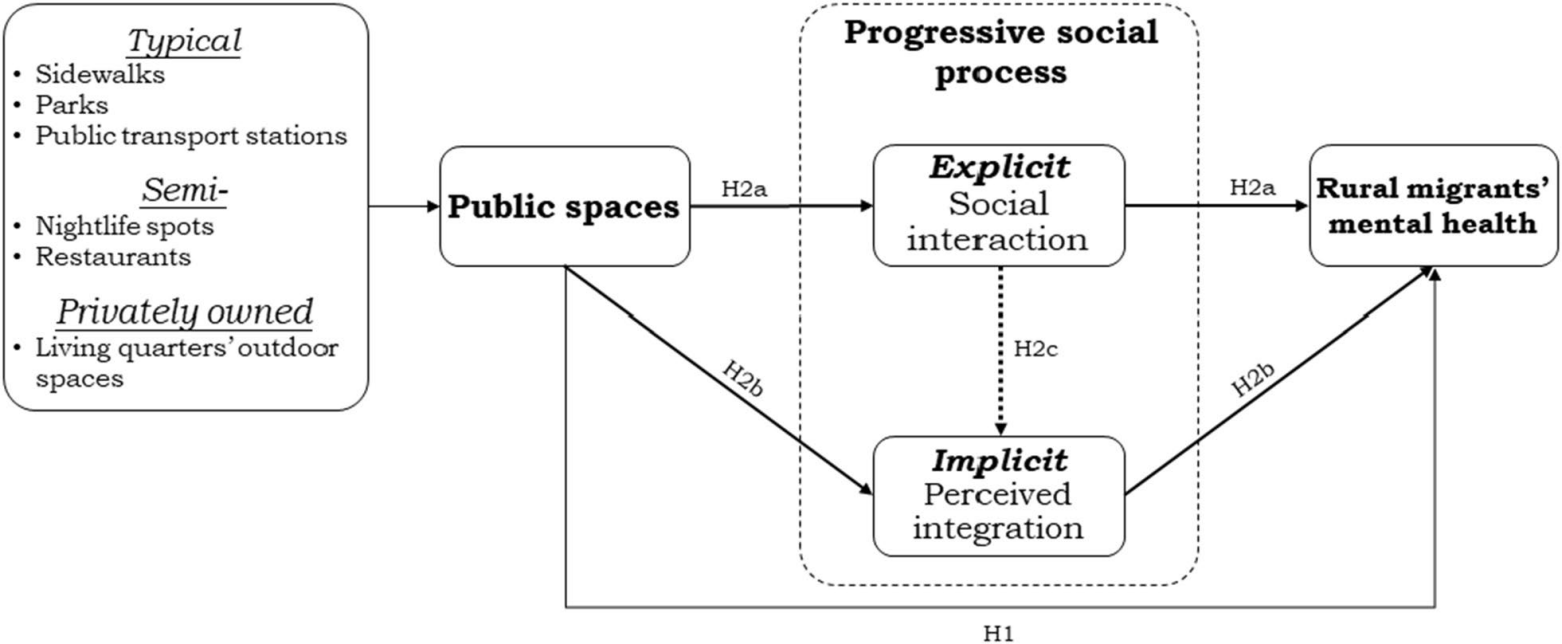
Research framework

Addressing this gap, our study proposes a theoretical framework to examine the impact of diversified public spaces on the mental health of rural migrants in urban China. It incorporates both explicit (social interaction) and implicit (perceived integration) mediators, detailing their sequential roles in the progressive social process to enrich our understanding of the intrinsic link between public spaces and mental health (Fig. 1). This adds to the knowledge needed to construct socially friendly public spaces essential for promoting inclusive urbanization.

## Methods

### Study area and data source

We chose Wuhan as the study area for three reasons. First, Wuhan is undergoing rapid urbanization, like Beijing, Shanghai, Guangzhou, and Shenzhen, etc., to be one of the major migrant-receiving cities in China. According to the Seventh National Population Census of China, in 2020, the migrant population in Wuhan was recorded at 3.95 million, constituting 32.0% of the city’s total population, which surpasses the national average of 26.6% [2]. Second, at the 2018 International Placemaking Workshop conference, Wuhan advocated for the enhancement of public spaces to promote equitable social and healthy habitat environments. Third, Wuhan’s 14th Five-Year Plan intensifies initiatives to build a healthy-oriented city by equalizing public service infrastructures, aiming to reduce health disparities between urban locals and rural migrants.

**Fig. 2:**
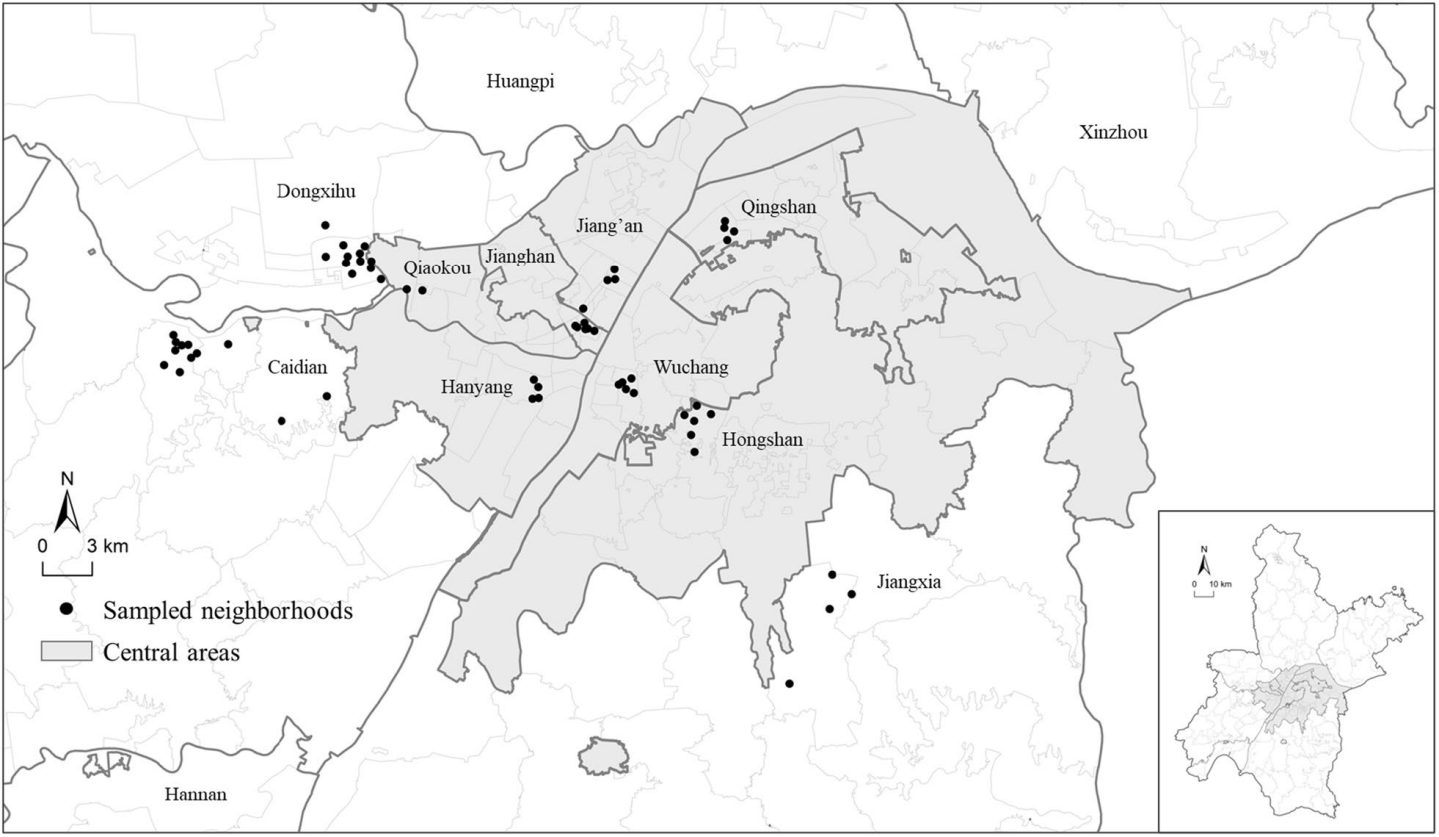
Location of study area

The data was collected in Wuhan, China, from September to November 2018 using a multi-stage stratified sampling approach. The Ethics Committee of Wuhan University approved the study. Participants were informed of the survey’s purpose, promised confidentiality, and provided consent prior to commencing before beginning. We adopted a targeted sampling by selecting subdistricts (*Jiedao*s) across seven central and three suburban districts. Next, we conducted a random sampling of neighborhoods and their associated rural migrants within each subdistrict, covering 60 neighborhoods and yielding 716 valid responses (Fig. 2). We controlled the gender balance to support sample validity. Migrants were defined as adults over 18 years old who had lived in Wuhan for at least six months without local household registration.

### Measurement of variables

#### Mental health

Rural migrants’ mental health was assessed using the 12-item General Health Questionnaire (GHQ-12) [50]. The respondents rated six positive (able to concentrate, playing a useful part, capable of making decisions, able to enjoy day-to-day activities, able to face problems, and feeling reasonably happy) and six negative items (loss of sleep over worry, felt constantly under strain, couldn’t overcome difficulties, feeling unhappy and depressed, losing confidence, and thinking of self as worthless) each on a five-point Likert scale from 1 (never) to 5 (always) [51]. We inversed negative items and aggregated 12 items (ranging from 12 to 60) to indicate migrants’ mental health, with a higher total score suggesting better mental health. Cronbach’s $\alpha$ was 0.73, showing strong internal consistency.

#### Three types of public spaces

In migrant health-related research, categorizing public spaces as typical, semi-, or privately owned is essential in response to the ‘*Spaces of Encounter*’ theory [13, 29]. This categorization acknowledges that the definition of public space is not universal across various groups. Historically, public spaces dominated by the native elite have inadvertently excluded vulnerable groups, including rural migrants, women, and the elderly [31]. Such monopolization may result in disparities in the interactions and health outcomes between the general population and rural migrants. Recognizing these disparities in ‘liminality’ among demographics and the ‘openness’ of spaces is crucial to understanding the specific health impacts on migrant populations. Therefore, our analysis focuses on these three public space categories, characterized by varied access challenges.

***Typical public spaces*** are characterized by their unrestricted accessibility [52]. Our study covers three typical public spaces: sidewalks, parks, and public transport stations (e.g., bus and subway stations).

***Semi-public spaces*** are tailored to specific demographic groups, with individuals often selected based on their interests or socioeconomic backgrounds [10, 52]. Our study covers nightlife spots (e.g., nightclubs, pubs, KTV) and restaurants (e.g., economical and chain restaurants). We specified an 800 m buffer zone as the neighborhood to measure typical and semi-public space indicators, aligning with Wuhan’s Planning Action for 15-Minute Community Life Circle in 2018. Sidewalk proportion was computed by dividing the neighborhood’s sidewalk length by the total road length (0–1). Other public space indicators were measured by density, calculated as the number of facilities per square kilometer using points of interest (POIs) data obtained from Gaode Map APIs (https://www.amap.com/).

**Fig. 3:**
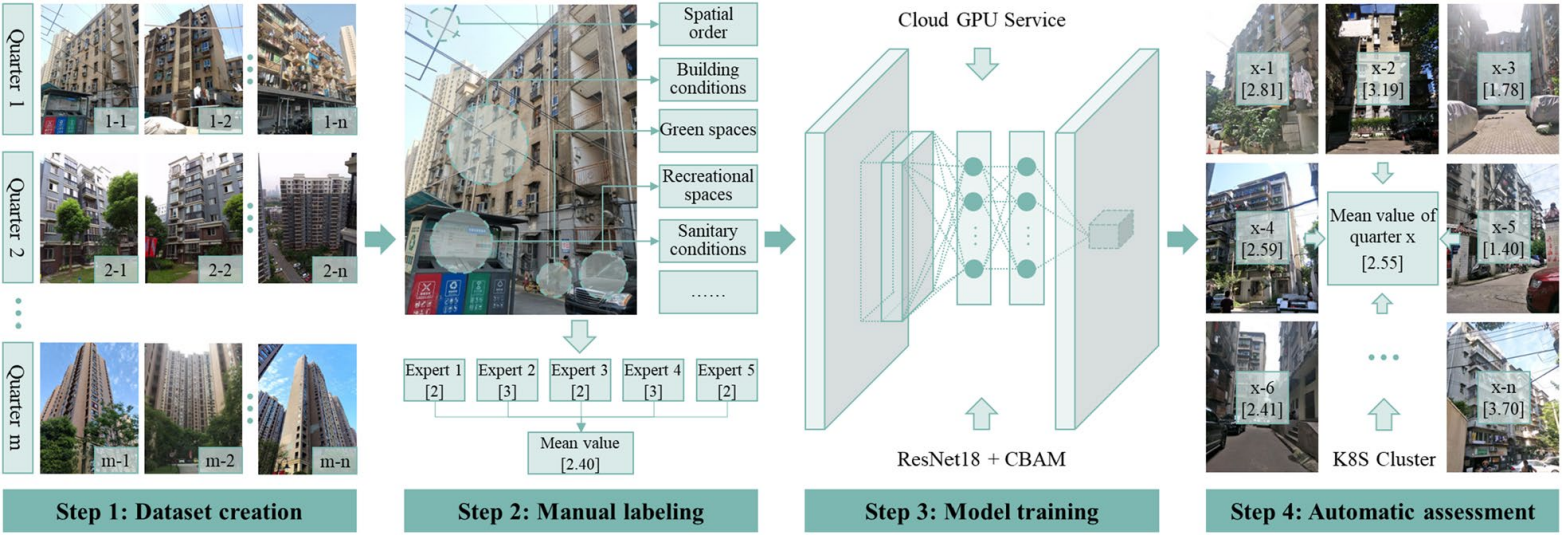
Physical quality of privately owned public spaces evaluated by deep learning

***Privately owned public spaces*** typically within the neighborhoods’ living quarters, restrict access to residents outside the neighborhoods [10]. We evaluated these spaces, focusing on their quality features, including both physical and social aspects. Physical-spatial quality, including aesthetic and functional appeal, was assessed using Anjuke (https://wuhan.anjuke.com/) image data with deep learning image recognition methods (Fig. 3). Detailed methodology is provided in Footnote^1^. Social-spatial quality was derived from residents’ positive feedback on neighborhoods’ spatial and governance issues, analyzed using Wuhan City Message Board (http://liuyan.cjn.cn/) text data and the Natural Language Processing API of Baidu AI Open Platform. Process details are available in Footnote^2^. Higher scores for physical- (1–5) and social-spatial (0–1) quality indicated favorable conditions. These public spaces, as sites of daily activities,  have been a focal point for research on health improvements through government interventions [53].

#### Social mediators

We focused on two mediators: social interaction and perceived integration. We measured social interaction based on the frequency of four activities from questionnaires, including (1) greeting/chatting with neighbors, (2) playing chess/cards/mahjong with neighbors, (3) having dinner/a walk with neighbors, and (4) helping each other with neighbors [36, 37]. This measurement covers all common spectrums of interactions that occur within neighborhoods, not just those confined to public spaces. While interactions in public spaces are crucial, we contend that the foremost goal in developing inclusive neighborhoods and social-supportive infrastructures lies in enabling widespread interaction, meriting significant emphasis. This approach to measuring social interaction is widely used in studies on public spaces and health [36, 43]. Each item was answered on a five-point Likert scale (1 = never, 5 = often) (Cronbach’s $\alpha$=0.74) (< 0.70). The social interaction score was derived by averaging four items.

Based on existing research [36, 41, 42], we measured migrants’ perceived integration by employing six questions: (1) ‘I have a strong sense of belonging to my community’, (2) ‘I have a strong emotional connection with my neighbors’, (3) ‘I trust my neighbors who are locals’, (4) ‘I would like to be friends with locals’, (5) ‘I feel that locals do not like me’, (6) ‘I feel that locals discriminate against me’, answering with a five-point Likert scale (1 = strongly disagree, 5 = completely agree). The final integration score was calculated by inverting the scale for the last two negative items and then averaging them with the scores of the remaining four items, with a Cronbach’s α of 0.73. Detailed information can be found in Additional file 1: Tables S1, S2.

#### Control variables

Individual socio-demographics are well-documented to be associated with mental health [3]. We set a series of control variables, including age, gender, education, and personal annual income (Table 1).

**Table 1:** Description of variables

Variables	Mean (SD)/Proportion
Dependent variable	
Mental health (12–60)	46.42 (5.03)
Independent variables	
Typical public spaces	
Sidewalks (0–1)	0.40 (0.14)
Parks (num./km^2^)	1.10 (1.25)
Public transport stations (num./km^2^)	5.60 (3.48)
Semi-public spaces	
Nightlife spots (num./km^2^)	3.73 (3.55)
Restaurants (num./km^2^)	139.95 (105.21)
Privately owned public spaces	
Physical-spatial quality (1–5)	2.82 (0.79)
Social-spatial quality (0–1)	0.18 (0.12)
Social mediators	
Social interaction (1–5)	2.38 (0.81)
Perceived integration (1–5)	3.57 (0.45)
Control variables	
Age (%)	
18–40	67.32
≥ 41	32.68
Gender (%)	
Male	46.23
Female	53.77
Education (%)	
High school or below	44.41
College or above	55.59
Personal annual income (%)	
< 50,000 CNY	48.74
≥ 50,000 CNY	51.26
N	716

### Statistical analysis

Multilevel structural equation modeling was employed to examine the intervening social mechanism linking public spaces to the mental health of rural migrants. This method was selected for two reasons. Firstly, it is suitable for the hierarchical data structure where individuals are nested within neighborhoods. Secondly, it can run complex models by including multiple predictors, mediators, and the outcome [54].

In Stata 16.0, we first calculated the intraclass correlation coefficient (*ICC*) using the null model of mental health [55]. The *ICC* value was 0.209, indicating that the neighborhood-level factors explained 20.9% of the variance in mental health. This result highlights the imperative of employing multilevel models. Subsequently, we tested the research framework (Fig. 1) using Multilevel Generalized Structural Equation Modeling (MGSEM), necessitated by the dichotomous and multicategorical nature of our variables. The ‘*nlcom*’ command facilitated the computation of direct, indirect, and total effects. Unlike traditional SEM, MGSEM primarily uses the Akaike Information Criterion (*AIC*), Bayesian Information Criterion (*BIC*), and log-likelihood to evaluate the models’ fit goodness [56]. Smaller *AIC* and *BIC *values, along with larger log-likelihood, signify a superior model fit. The mean Variance Inflation Factor (*VIF*) was 1.68 (< 3.0), indicating no severe issues with multicollinearity among variables. To enable a direct comparison of the impact of various public spaces on the mental health of rural migrants, we standardized all continuous variables in the MGSEM analysis.

## Results

### Descriptive statistics

The respondents’ demographic profiles and public space attributes are shown in Table 1. The majority of respondents were aged 18–40 (67.32%), with a slightly higher proportion of females (53.77%) compared to males (46.23%). Over half held a bachelor’s degree or above (55.59%) and reported an annual income of at least 50,000 CNY (51.26%).

Rural migrants reside in neighborhoods characterized by an average density of 1.10 parks, 5.60 public transport stations, 3.73 nightlife spots, and 139.95 restaurants per square kilometer. Access to typical public spaces, notably parks and walking paths, is limited, with only 40% of walking paths being accessible. In contrast, access to semi-public spaces like nightlife spots and restaurants is deemed moderate. Additionally, both physical- (2.82) and social- (0.18) spatial quality scores were observed to be below the midpoint of their respective scales (3.0 and 0.5), reflecting the poor and disadvantaged conditions of privately owned public spaces.

Regarding social factors, the study found that rural migrants exhibited a relatively low average social interaction score (2.38) yet a notably high perceived integration score (3.57), both benchmarked against a scale midpoint of 3.0. The mean mental health score among participants was 46.42, marginally below the neutral benchmark of 47, indicating a modestly unsatisfactory level of mental well-being within the sample. These findings underscore the potential for targeted interventions within public spaces to enhance mental health outcomes among rural migrants.

### Model fitting parameters

We conducted five different models to evaluate the models’ fit goodness (see Table 2). The fitting parameters of Model 1 (the direct effects of public spaces) and Model 2 (adding control variables) showed slight differences, demonstrating the significant influence of public spaces on mental health, even after adjusting for individual socio-demographic characteristics. Compared to Model 3 (considering parallel mediation of social interaction and perceived integration) and Model 4 (considering serial mediation of the influence of social interaction on perceived integration), Model 5 (full model) exhibited superior goodness of fit, with the lowest *AIC* (5865.003) and the second lowest *BIC* (6070.756), alongside the highest log-likelihood (2887.502), indicating the need for considering both parallel and serial mediation mechanisms to fully capture the dynamics at play. Furthermore, the comparison between Models 3 (parallel mediation) and 4 (serial mediation) with Model 5 indicated a more substantial explanatory contribution from the serial mediation pathway. Consequently, further analyses will prioritize Model 5 for in-depth examination.

**Table 2:** Model fitting parameters

	*AIC*	*BIC*	*Log-likelihood*
Model 1 (PS → MH)	1954.569	2000.306	− 967.285
Model 2 (PS + CV → MH)	1943.252	2007.284	− 957.626
Model 3 (PS + CV → SI/PI → MH)	5923.887	6093.114	− 2924.944
Model 4 (PS + CV → SI → PI → MH)	5881.992	6019.161	− 2910.996
Model 5 (full model)	5865.003	6070.756	− 2887.502

*PS* public spaces, *MH* mental health, *CV* control variables, *SI* social interaction, *PI* perceived integration

### Direct effects of diverse public spaces on mental health

**Fig. 4:**
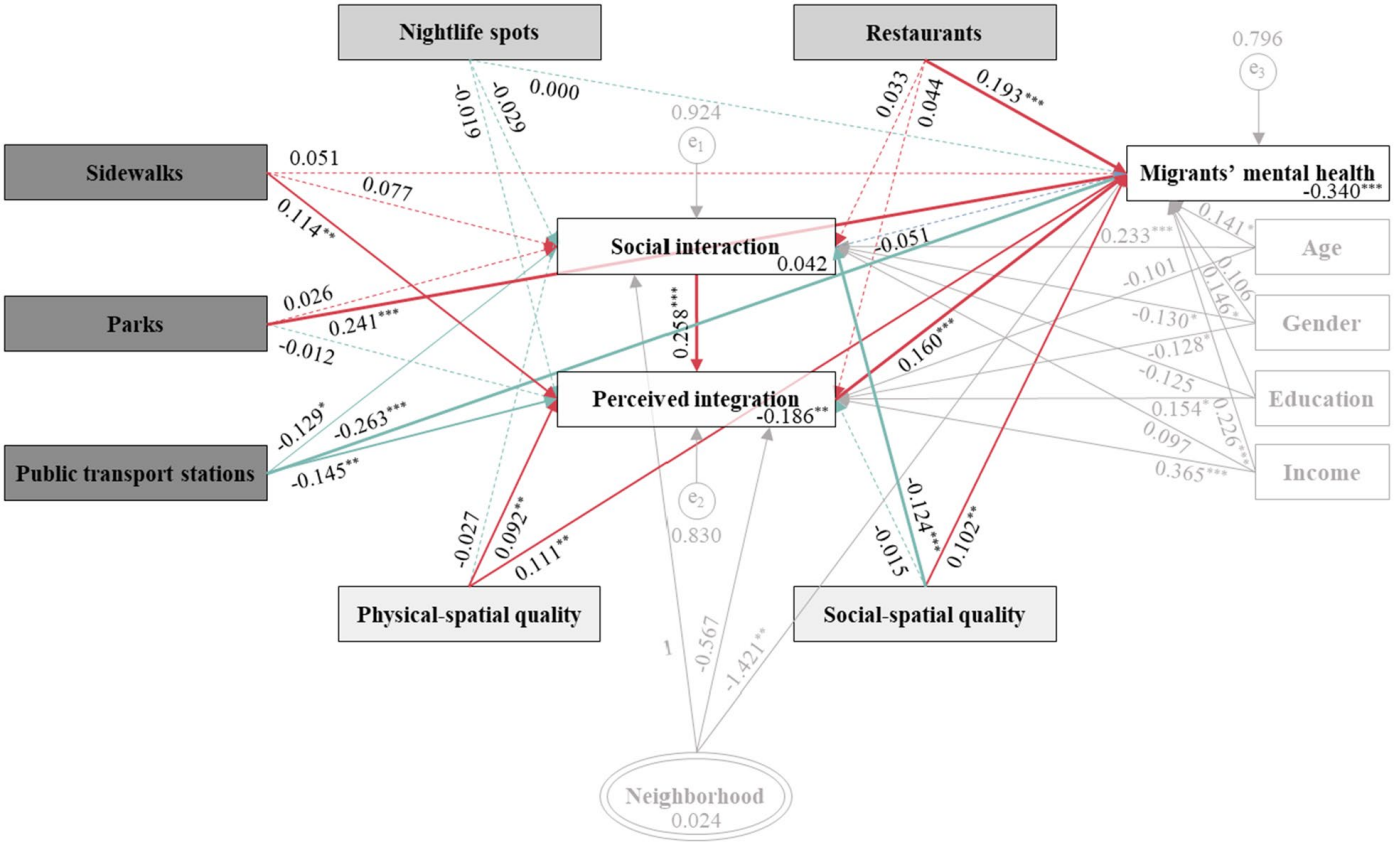
Multilevel Generalized Structural Equation Modeling (MGSEM) results. **a**. Variables in the dark, medium, and light gray boxes are typical, semi-, and privately owned public spaces, respectively; **b**. Red and green arrows indicate positive and negative effects; **c**. Arrow thickness reflects significance level: *P* < 0.10 (*), *P* < 0.05 (**), *P* < 0.01 (***); **d**. All continuous variables are standardized

Figure 4 and Additional file 1: Table S3 illustrate the full MGSEM (Model 5) results. Our findings indicated that public spaces directly impacted rural migrants’ mental health, albeit with positive or negative outcomes. This influence varied significantly depending on whether the public spaces were typical, semi-, or privately owned, confirming **H1.** For typical public spaces, the presence of additional parks within a neighborhood significantly enhanced migrants’ mental health (*β* = 0.241, *P* < 0.01). In contrast, an increased density of public transport stations was inversely related to the mental health outcome (*β* = − 0.263, *P* < 0.01). Interestingly, the sidewalk proportion showed a positive yet statistically insignificant association with mental health (*β* = 0.051). In the case of semi-public spaces, our findings indicated a significant positive relationship between restaurant density and mental health (*β* = 0.193, *P* < 0.01), with nightlife spots demonstrating minimal impact. Concerning privately owned public spaces, both physical- and social-spatial quality significantly enhanced rural migrants’ mental health, exhibiting coefficients of 0.111 and 0.102, respectively, statistically significant at *P* < 0.05. These results highlight the nuanced roles that various types of public spaces play in influencing mental health among rural migrants, emphasizing the importance of thoughtful public space design and governance.

### Social capacity-building pathways

We employed the MGSEM method to probe the intermediary effects of social factors, specifically social interaction and perceived integration (Fig. 4), as well as the direct, indirect, and overall effects of public spaces on the mental health of rural migrants in each pathway.

Table 3 displays the significance of each path in the MGSEM, accompanied by 95% confidence intervals (95% CI). A path is deemed significant if its 95% CI does not include 0; otherwise, it is considered non-significant. In examining the role of social interaction, our analysis did not yield sufficient evidence to support the hypothesis that public spaces facilitate social interaction, subsequently improving mental health (parallel mediation of social interaction: ‘public spaces → social interaction → mental health’). Most public space elements seemed to have no connection with migrants’ social interaction within neighborhoods. Furthermore, although specific public spaces, such as public transport stations and the social-spatial quality of living quarters, might slightly influence rural migrants’ social interactions, these effects did not significantly impact their mental health. This lack of impact could be attributed to the absence of a significant relationship between social interaction and mental health. Consequently, **H2a** is rejected.

**Table 3:** Effects of public spaces on rural migrants’ mental health in full model

Pathways	Direct effect	Indirect effect	Total effect
	β (95% CI)	β (95% CI)	β (95% CI)
Typical public spaces			
SW → SI → MH		− 0.004 (− 0.012, 0.004)	
SW → PI → MH		0.018^**^ (0.001, 0.035)	
SW → SI → PI → MH		0.003 (− 0.001, 0.008)	
SW → MH	0.051 (− 0.064, 0.165)	0.017^*^ (0.000, 0.035)	0.068 (− 0.051, 0.187)
PK → SI → MH		− 0.001 (− 0.007, 0.004)	
PK → PI → MH		− 0.002 (− 0.015, 0.011)	
PK → SI → PI → MH		0.001 (− 0.003, 0.005)	
PK → MH	0.241^***^ (0.133, 0.349)	− 0.002 (− 0.016, 0.011)	0.239^***^ (0.126, 0.352)
PTS → SI → MH		0.007 (− 0.005, 0.018)	
PTS → PI → MH		− 0.023^**^ (− 0.045, − 0.002)	
PTS → SI → PI → MH		− 0.005^*^ (− 0.012, 0.001)	
PTS → MH	− 0.263^***^ (− 0.408, − 0.118)	− 0.022^*^ (− 0.045, 0.002)	− 0.285^***^ (− 0.435, − 0.135)
Semi-public spaces			
NLS → SI → MH		0.001 (− 0.005, 0.007)	
NLS → PI → MH		− 0.005 (− 0.021, 0.012)	
NLS → SI → PI → MH		− 0.001 (− 0.006, 0.004)	
NLS → MH	0.000 (− 0.121, 0.121)	− 0.004 (− 0.021, 0.012)	− 0.004 (− 0.130, 0.121)
RES → SI → MH		− 0.002 (− 0.010, 0.006)	
RES → PI → MH		0.007 (− 0.014, 0.028)	
RES → SI → PI → MH		0.001 (− 0.005, 0.008)	
RES → MH	0.193^**^ (0.032, 0.354)	0.007 (− 0.014, 0.028)	0.200^**^ (0.032, 0.367)
Privately owned public spaces			
PSQ → SI → MH		0.001 (− 0.004, 0.006)	
PSQ → PI → MH		0.015^**^ (0.000, 0.029)	
PSQ → SI → PI → MH		− 0.001 (− 0.005, 0.003)	
PSQ → MH	0.111^**^ (0.012, 0.211)	0.015^**^ (0.000, 0.030)	0.127^**^ (0.024, 0.229)
SSQ → SI → MH		0.006 (− 0.004, 0.016)	
SSQ → PI → MH		− 0.002 (− 0.015, 0.010)	
SSQ → SI → PI → MH		− 0.005^**^ (− 0.010, − 0.000)	
SSQ → MH	0.102^**^ (0.002, 0.203)	− 0.001 (− 0.016, 0.014)	0.101^*^ (− 0.003, 0.205)

^*^P < 0.10, ** *P* < 0.05, *** *P* < 0.01

*SI* social interaction, *PI* perceived integration, *MH* mental health, *SW* sidewalks, *PK* parks, *PTS* public transport stations, *NLS* nightlife spots, *RES* restaurants, *PSQ* physical-spatial quality, *SSQ* social-spatial quality

Furthermore, the results suggest that perceived integration serves as a critical mediator. Specifically, enhanced accessibility to sidewalk facilities demonstrated a positive indirect relationship with mental health, mediated by strong perceived integration (*β* = 0.018, 95% CI [0.001, 0.035]). Additionally, the physical-spatial quality of neighborhood quarters significantly improved the mental health of rural migrants through elevated perceived integration (*β* = 0.015, 95% CI [0.000, 0.029]). Conversely, the accessibility of public transport stations, measured by facility density, negatively impacted mental health by diminishing perceived integration (*β* = − 0.023, 95% CI [− 0.045, − 0.002]). Indirect effects stemming from other public spaces did not achieve statistical significance. These findings corroborate **H2b**, suggesting a parallel mediating role for perceived integration (‘public spaces → perceived integration → mental health’).

Given the significant positive correlation between social interaction and perceived integration among rural migrants (*β* = 0.258, *P* < 0.01) (Fig. 4), we examined the presence of serial mediation effects. The findings reveal a progressive social process linking ‘public spaces’ with ‘social interaction’, ‘perceived integration’, and ultimately ‘mental health’. Specifically, public transport stations (*β* = -0.005, 95% CI [− 0.012, 0.001]) and social-spatial quality within neighborhoods (*β* = − 0.005, 95% CI [− 0.010, − 0.000]) both exhibited negative indirect impacts on mental health through the ‘social interaction → perceived integration’ pathway. Serial mediating effects associated with other public spaces failed to reach statistical significance. Overall, **H2c** is supported: certain public spaces can enhance rural migrants’ mental health by encouraging meaningful social interactions and ensuing sound integration.

## Discussions

Focusing on Wuhan, a city experiencing a remarkable surge in the migrant population, this study examined the impacts of different types of public spaces (i.e., typical, semi-, and privately owned) on rural migrants’ mental health and revealed the progressive mediating process of social factors. We identify four interesting findings that are particularly novel and worth special attention.

### Main findings

Our findings corroborate previous studies suggesting that well-established public spaces can promote the mental health of rural migrants in urban China. In particular, rural migrants reported fewer mental health problems when they resided in neighborhoods characterized by high-density parks [24], restaurants [26], and favorable physical- and social-spatial qualities [24]. Nonetheless, certain conclusions of our study are inconsistent with the existing body of literature. For instance, individuals who migrated from rural to urban areas, residing in neighborhoods with densely located public transport stations, exhibited poorer mental health. This finding lends support to the ongoing discourse concerning the uncertain health effects associated with public transportation [25]. Although residing in proximity to highly accessible public transport stations may confer certain mental health benefits, these advantages do not fully counteract the negative impacts of air pollution, traffic noise, overcrowding, and other environmental stressors stemming from high population density and urbanization, particularly in China [57]. No evidence exists for a significant direct relationship between sidewalks and nightlife spots and the mental health of rural migrants in Wuhan. The limited daily walking activities of rural migrants curtail the potential mental health benefits of sidewalks, attributed to the extensive commuting distances [48]. Meanwhile, the heavy life and work burdens, coupled with economic constraints, reduce the possibility of frequent nightclub visits [58], leading to minimal mental health impacts. Typical, semi-, and privately owned public spaces directly influence the mental health of rural migrants. However, specific attributes of these spaces may exert either positive or negative effects on health.

Second, we extended the ‘*Spaces of Encounter*’ theory to differentiate between the ‘explicit’ (social interaction) and ‘implicit’ (perceived integration) social factors concerning their roles between three types of public spaces and migrants’ mental health. The MGSEM outcomes show that while certain public spaces may strengthen or weaken the frequency of social interaction among rural migrants, they do not necessarily have further impacts on their mental health. This finding stands in contrast to mainstream studies that overpraise the virtues of neighborly interactions for their health-promoting benefits [59]. Recent research has reflected on the diminishing returns of frequent social interaction on mental well-being [60]. This phenomenon is particularly evident among rural migrants in China, for whom social interactions are not always beneficial. A contributing factor may be the perceived intensification of social and economic disparities between rural migrants and urban locals resulting from increased social interactions [46]. This perception can exacerbate comparisons among different social groups, potentially deteriorating migrants’ mental health [61]. Moreover, as discussed in the literature review, negative externalities such as ‘free riding’(*Dabianche*), heightened return expectations, and participation in slavery may precipitate the involution and self-isolation of migrants’ interactions [62]. Such adverse effects can further erode their mental health.

Furthermore, we confirm the crucial mediating function of perceived integration among rural migrants. This insight extends the existing literature on the impact of social integration on mental health, derived from public spaces in various Chinese cities including Beijing [10], Shanghai [63], Guangzhou [3], and Shenzhen [34]. The study offers novel perspectives for rural migrants in Wuhan, illustrating how sidewalks contribute to positive health outcomes through the enhancement of perceived integration, rather than exerting a direct effect. Additionally, our analysis reveals that improved physical-spatial quality significantly promotes social integration, which in turn, supports the mental health of rural migrants. This effect is attributed to high-quality spaces affording greater access to opportunities for frequent and meaningful encounters, thereby cultivating deep-seated and solid social connections [64].

Last but not least, our study has demonstrated that the mental health benefits of social interaction depend on its conversion into meaningful integration. This serial social mechanism was validated in both typical and privately owned public spaces, yet not within semi-public spaces. Specifically, while the high-density of public transport stations, serving as transitional spaces, may engender fleeting social contact, it fails to promote profound perceived integration of intergroups, resulting in decreased mental health. This observation aligns with earlier studies [43]. Additionally, the mediating role of social factors between social-spatial quality and mental health presented as negative. A plausible reason is that in areas afflicted by social dilemmas or governance disputes, rural migrants and urban dwellers seldom engage in dialogue to address challenges, thereby inhibiting integration and diminishing health benefits. Moreover, the dominance of local elites over rural migrants on online message boards may distort the representation of the issues, contributing to biased perspectives. Factors such as the digital divide, educational disparities, and divergent propensities for community involvement are likely to contribute to this disparity [65].

In summary, our study suggests that the mental consequences of social factors depend on rural migrants’ unrestricted access to public spaces. Significant social effects were observed in both typical and privately owned public spaces, albeit with reduced functionality in semi-public spaces. Such findings challenge the prevailing belief that social interactions intensify as the publicness of spaces diminishes, as traditionally intended for the general population [13]. In China, *typical public spaces* offering unrestricted access promote inclusivity and free interaction among rural migrants, thus affecting their mental health positively. *Privately owned public spaces*, predominantly situated within gated communities, acting as ‘containers’ for a socioeconomically diverse mix of migrant and local residents, facilitating enduring and beneficial intergroup relations that enhance mental health. The negligible social function of *semi-public spaces* can be ascribed to factors such as crowd preferences, financial limitations, exclusion, and discrimination within these environments [66]. As a result, high-consumption entertainment venues, including nightclubs and upscale restaurants, are less favored by some rural migrants, especially those with low incomes. This serves as a reminder that the so-called ‘publicness’ includes both visible and invisible barriers, posing significant challenges for vulnerable groups, such as rural migrants. These challenges include not only physical access limitations but also socio-economic barriers that exacerbate isolation and hinder the integration of rural migrants into urban neighborhoods.

### Strengths and limitations

From a theoretical perspective, this study enriches the existing body of literature by clarifying the progressive social process underlying public spaces and rural migrants’ mental health, going beyond isolated analyses of social factors. Specifically, we detail the mechanism by which public places facilitate explicit social interaction, resulting in implicit perceived integration, and subsequently impacting the mental health of rural migrants. This serial social functional mechanism contributes to the existing corpus of knowledge on migrant health. Methodologically, this research integrates traditional geospatial data with emerging image and text data for computing neighborhood environmental indicators using deep learning techniques. This approach advances the micro-environment assessment system in a technology-driven era.

Despite the aforementioned strengths, this study acknowledges several limitations. Firstly, although this cross-sectional study elucidates the interplay between various factors, it does not ascertain causation. Future research should explore causality through longitudinal studies and natural experiments. Secondly, the study primarily examines the existence of public spaces, underestimating the extent of migrants’ engagement with these areas. Public spaces’ contribution to health promotion extends beyond mere existence, including the utilization, occupation, and activity within these spaces as crucial health determinants. Future studies should delve into the impact of public spaces on mental health, considering both the presence and the engagement with these spaces.

### Policy implications

Our empirical findings render the following policy implications. Creating migrant-friendly neighborhood with accessible parks and restaurants is of great significance to improving rural migrants’ mental health. Moreover, the development of high-quality privately owned public spaces, with a focus on landscapes, facilities, and sanitation, is vital for supporting migrants’ health in these areas. Public spaces that are well-established are essential for thriving neighborhoods, offering significant social benefits. Our study suggests that mere social interaction is insufficient for improving migrants’ mental health; integration is imperative. Hence, initiatives that promote deep and sustained interaction between migrants and local residents in public spaces are essential. Engaging in cultural and recreational activities in public spaces enable meaningful interactions beyond transient contacts. Encouraging deep involvement in public spaces can markedly improve the mental health of rural migrants.

## Conclusions

Previous research based on the ‘*Spaces of Encounter*’ theory has reached a consensus that social interactions in public spaces yield positive social outcomes, such as promoting mental health. Using Wuhan as a case study, we employed Multilevel Generalized Structural Equation Modeling (MGSEM) to unpack the social capacity-building pathways occurring in associations between public spaces and rural migrants’ mental health based on multi-source data. Our research examined both explicit and implicit social intermediary mechanisms, contributing to existing literature and questioning prevailing assumptions. Our findings revealed that privately owned public spaces, characterized by higher physical- and social-spatial quality, exhibited more pronounced positive effects on migrants’ mental health than typical and semi-public spaces. Notably, the impact of social interaction on the mental health depended on the pathway to perceived integration, emphasizing the significance of cultivating meaningful interactions developing into deep-seated integration rather than superficial encounters. Specifically, typical and privately owned public spaces held significant social effects on the promotion of migrants’ mental health, whereas semi-public spaces exhibited limited social functions.

### Supplementary Information

**Additional file 1: Table S1.** Measurement of social interaction. **Table S2.** Measurement of perceived integration. **Table S3.** Multilevel Generalized Structural Equation Modeling (MGSEM) results.

## Data Availability

The data is not publicly available due to privacy or ethical restrictions. Requests to access the datasets should be directed to the corresponding author (hanbeicheng@tsinghua.edu.cn).

## Footnotes

1 *Physical-spatial quality measurement process*. (1) Dataset Creation: An image dataset was compiled from 7,895 residential quarters in Wuhan, sourced from Anjuke (https://wuhan.anjuke.com/). Image semantic segmentation was applied to identify environmental elements in the image, identifying valid images with clear building facades and surrounding facilities. A total of 99,567 valid images were captured. We randomly selected 1000 images from each district, for a total of 13,000 images as the labeled dataset. (2) Manual labeling: Five volunteers labeled images on the scoring platform. They evaluated each image in the labeled dataset on various parameters such as building conditions, recreational spaces, green spaces, sanitary conditions, and spatial order. The images were scored on a scale of 1–5, with the average score representing the label value. (3) Model training: The images in the labeled dataset were randomly divided into training and test sets (4:1). A regression model, incorporating a Convolutional Block Attention Module (CBAM) to ResNet18, was trained, leveraging Cloud GPU Service for computational support. The model achieved a high R^2^ of 0.94. (4) Automatic assessment: The trained model was deployed in a K8S Cluster, automatically evaluating the physical-spatial quality of 60 sampled quarters.

2 *Social-spatial quality measurement process***. **(1) Dataset Creation: 3836 message texts were scraped from the Wuhan City Message Board (http://liuyan.cjn.cn/) using the names of 60 sampled residential quarters as keywords. (2) Emotional computing: The Natural Language Processing API of the Baidu AI Open Platform was called to calculate the positive emotional score of each message (0–1). (3) Score Summarization: The average of all message values in each residential quarter was considered as the social-spatial quality score.

## References

[1] Fu X, Zhang K, Chen X, Chen Z. Report on National Mental Health Development in China (2019~2020). 2021.

[2] National Bureau of Statistics of China. Bulletin of the Seventh National Census (No.7). 2021.

[3] Liu Y, Zhang F, Wu F, Liu Y, Li Z (2017). The subjective wellbeing of migrants in Guangzhou, China: the impacts of the social and physical environment. Cities.

[4] Liu Z, Wang Y, Tao R (2013). Social capital and migrant housing experiences in urban China: a structural equation modeling analysis. Hous Stud.

[5] Zhang Y, You C, Pundir P, Meijering L (2023). Migrants’ community participation and social integration in urban areas: a scoping review. Cities.

[6] Zhang Z, Jia Z, Zhou Z (2022). Can urban green space cure homesickness? Case study on China poverty alleviation migrants in Anshun, Guizhou. Urban For Urban Green.

[7] Wen M, Fan J, Jin L, Wang G (2010). Neighborhood effects on health among migrants and natives in Shanghai, China. Heal Place.

[8] Yue Z, Li S, Jin X, Feldman MW (2013). The role of social networks in the integration of Chinese rural-urban migrants: a migrant-resident tie perspective. Urban Stud.

[9] Mouratidis K (2021). Urban planning and quality of life: a review of pathways linking the built environment to subjective well-being. Cities.

[10] Liu Z, Tan Y, Chai Y (2020). Neighbourhood-scale public spaces, inter-group attitudes and migrant integration in Beijing, China. Urban Stud.

[11] Chan KW (2010). The household registration system and migrant labor in China: notes on a debate. Popul Dev Rev.

[12] Jian IY, Luo J, Chan EHW (2020). Spatial justice in public open space planning: accessibility and inclusivity. Habitat Int.

[13] Piekut A, Valentine G (2017). Spaces of encounter and attitudes towards difference: a comparative study of two European cities. Soc Sci Res.

[14] Rook KS (2015). Social networks in later life: weighing positive and negative effects on health and well-being. Curr Dir Psychol Sci.

[15] Dai M, He S (2024). Social mix and subjective wellbeing in Chinese urban neighborhoods: exploring the domino effects of social capital through multilevel serial mediation analysis. Habitat Int.

[16] Liu Y, Wang R, Lu Y, Li Z, Chen H, Cao M (2020). Natural outdoor environment, neighbourhood social cohesion and mental health: using multilevel structural equation modelling, streetscape and remote-sensing metrics. Urban For Urban Green.

[17] Suglia SF, Duarte CS, Sandel MT (2011). Housing quality, housing instability, and maternal mental health. J Urban Heal.

[18] Zhang L, Zhang R, Huang Y, Liu L (2022). Urban physical examination evaluation index system research and practice towards integrated community development. Planners.

[19] Yao Y, Xu C, Yin H, Shao L, Wang R (2022). More visible greenspace, stronger heart? Evidence from ischaemic heart disease emergency department visits by middle-aged and older adults in Hubei, China. Landsc Urban Plan.

[20] Chen J, Zhang Z, Long Y (2020). Strategies for improving the quality of urban street space oriented to promoting public health: perspective from spatial quality. City Plan Rev.

[21] Fan Z, Zhang F, Loo BPY, Ratti C (2023). Urban visual intelligence: uncovering hidden city profiles with street view images. Proc Natl Acad Sci USA.

[22] Gao Z, Li Y, Jiang W, Gu J (2023). Research on spatial identification and influencing factors of urban governance problems: an exploration based on city message board data of Wuhan. Hum Geogr.

[23] Berke EM, Gottlieb LM, Moudon AV, Larson EB (2007). Protective association between neighborhood walkability and depression in older men. J Am Geriatr Soc.

[24] Zhu W, Wang J, Qin B (2021). Quantity or quality? Exploring the association between public open space and mental health in urban China. Landsc Urban Plan.

[25] Honold J, Beyer R, Lakes T, van der Meer E (2012). Multiple environmental burdens and neighborhood-related health of city residents. J Environ Psychol.

[26] Finlay J, Esposito M, Tang S, Gomez-Lopez I, Sylvers D, Judd S (2020). Fast-food for thought: Retail food environments as resources for cognitive health and wellbeing among aging Americans?. Heal Place.

[27] Francis J, Wood LJ, Knuiman M, Giles-Corti B (2012). Quality or quantity? Exploring the relationship between public open space attributes and mental health in Perth, Western Australia. Soc Sci Med.

[28] Mehta V (2014). Evaluating public space. J Urban Des.

[29] Matejskova T, Leitner H (2011). Urban encounters with difference: the contact hypothesis and immigrant integration projects in eastern Berlin. Soc Cult Geogr.

[30] Haase D, Jänicke C, Wellmann T (2019). Front and back yard green analysis with subpixel vegetation fractions from earth observation data in a city. Landsc Urban Plan.

[31] Gholamhosseini R, Pojani D, Mateo Babiano I, Johnson L, Minnery J (2019). The place of public space in the lives of Middle Eastern women migrants in Australia. J Urban Des.

[32] Maas J, van Dillen SME, Verheij RA, Groenewegen PP (2009). Social contacts as a possible mechanism behind the relation between green space and health. Heal Place.

[33] Oldenburg R, Brissett D (1982). The third place. Qual Soc.

[34] Yang M, Dijst M, Faber J, Helbich M (2020). Using structural equation modeling to examine pathways between perceived residential green space and mental health among internal migrants in China. Environ Res.

[35] Wang W, Fan C (2012). Migrant workers’ integration in urban China: experiences in employment, social adaptation, and self-identity. Eurasian Geogr Econ.

[36] Sugiyama T, Leslie E, Giles-Corti B, Owen N (2008). Associations of neighbourhood greenness with physical and mental health: do walking, social coherence and local social interaction explain the relationships?. J Epidemiol Commun Health.

[37] Wilkerson A, Carlson NE, Yen IH, Michael YL (2012). Neighborhood physical features and relationships with neighbors: does positive physical environment?. Environ Behav.

[38] Sharmeen F, Arentze T, Timmermans H (2014). Dynamics of face-to-face social interaction frequency: role of accessibility, urbanization, changes in geographical distance and path dependence. J Transp Geogr.

[39] Kawachi I, Kennedy BP, Glass R (1999). Social capital and self-rated health: a contextual analysis. Am J Public Health.

[40] Berkman LF, Glass T, Brissette I, Seeman TE (2000). From social integration to health: durkheim in the new millennium. Soc Sci Med.

[41] McMillan DW, Chavis DM (1986). Sense of community: a definition and theory. J Commun Psychol.

[42] Fan Y, Das KV, Chen Q (2011). Neighborhood green, social support, physical activity, and stress: assessing the cumulative impact. Heal Place.

[43] Liu Z, Wang X, Ma J (2020). The influence of public spaces on neighborhood social interaction in transitional urban Beijing: comparing local residents and migrants. Sci Geogr Sin.

[44] Marmot M (2005). Social determinants of health inequalities. Lancet.

[45] Holland C, Clark A, Katz J, Peace S (2007). Social interactions in urban public places.

[46] Hu R, Chen S (2012). Social factors influencing peasant workers’ mental health. Chinese J Sociol.

[47] Villalonga-Olives E, Kawachi I (2017). The dark side of social capital: a systematic review of the negative health effects of social capital. Soc Sci Med.

[48] Ouyang W, Wang B, Tian L, Niu X (2017). Spatial deprivation of urban public services in migrant enclaves under the context of a rapidly urbanizing China: an evaluation based on suburban Shanghai. Cities.

[49] Wen M, Wang G (2009). Demographic, psychological, and social environmental factors of loneliness and satisfaction among rural-to-urban migrants in Shanghai. China Int J Comp Sociol.

[50] Goldberg DP, Hillier VF (1979). A scaled version of the general health questionnaire. Psychol Med.

[51] Donath S (2001). The validity of the 12-item general health questionnaire in Australia: a comparison between three scoring methods. Aust N Z J Psychiatry.

[52] Wang X, Liu Z (2022). Neighborhood environments and inclusive cities: an empirical study of local residents’ attitudes toward migrant social integration in Beijing, China. Landsc Urban Plan.

[53] Pfeiffer D, Cloutier S (2016). Planning for happy neighborhoods. J Am Plan Assoc.

[54] Goldstein H (2011). Multilevel statistical models.

[55] Hox JJ, Maas CJM (2001). The accuracy of multilevel structural equation modeling with pseudobalanced groups and small samples. Struct Equ Model.

[56] Huang B, Xiao T, Grekousis G, Zhao H, He J, Dong G (2021). Greenness-air pollution-physical activity-hypertension association among middle-aged and older adults: evidence from urban and rural China. Environ Res.

[57] Zhang L, Zhou S, Kwan MP, Shen M (2021). Assessing individual environmental exposure derived from the spatiotemporal behavior context and its impacts on mental health. Heal Place.

[58] Li J, Rose N (2017). Urban social exclusion and mental health of China’s rural-urban migrants—a review and call for research. Heal Place.

[59] Wen M, Zheng Z, Niu J (2017). Psychological distress of rural-to-urban migrants in two Chinese cities: Shenzhen and Shanghai. Asian Popul Stud.

[60] Luo M, Macdonald B, Hülür G (2022). Not, “the more the merrier”: diminishing returns to daily face-to-face social interaction frequency for well-being in older age. J Gerontol Ser B.

[61] Valentine G (2008). Living with difference: reflections on geographies of encounter. Prog Hum Geogr.

[62] Woolcock M (1998). Social capital and economic development: toward a theoretical synthesis and policy framework. Theory Soc.

[63] Ta N, Kwan MP, Lin S, Zhu Q (2021). The activity space-based segregation of migrants in suburban Shanghai. Appl Geogr.

[64] Coley RL, Kuo FE, Sullivan WC (1997). Where does community grow? The social context created by nature in urban public housing. Environ Behav.

[65] Kahila-Tani M, Kytta M, Geertman S (2019). Does mapping improve public participation? Exploring the pros and cons of using public participation GIS in urban planning practices. Landsc Urban Plan.

[66] Hickman P (2013). “Third places” and social interaction in deprived neighbourhoods in Great Britain. J Hous Built Environ.

