# Supplementary material for: Revisiting the impact of public spaces on the mental health of rural migrants in Wuhan: an integrated multi-source data analysis
Source: Int J Health Geogr. 2024 Mar 7;23:7. doi: 10.1186/s12942-024-00365-8 (PMC10918943; doi:10.1186/s12942-024-00365-8)
Supplement: Supplementary file 1 — Additional file 1: Table S1. Measurement of social interaction. Table S2. Measurement of perceived integration. Table S3. Multilevel Generalized Structural Equation Modeling (MGSEM) results. [file 12942_2024_365_MOESM1_ESM.docx]

# Appendix

**Table S1** Measurement of social interaction.

| Questions (never = 1, rarely = 2, sometimes = 3, often = 4, always = 5) | Mean | SD | Min | Max |
| --- | --- | --- | --- | --- |
| How often do you greet or chat with your neighbors? | 2.41 | 1.29 | 1.00 | 5.00 |
| How often do you play chess, cards, or mahjong with your neighbors? | 1.99 | 1.21 | 1.00 | 5.00 |
| How often do you have dinner or a walk with your neighbors? | 2.48 | 0.90 | 1.00 | 5.00 |
| How often do you and your neighbors help each other (e.g., borrow tools, watch children, run errands)? | 2.63 | 0.86 | 1.00 | 5.00 |
| *Average score (Cronbach’s* $\alpha$*=0.74)* | 2.38 | 0.81 | 1.00 | 4.50 |

**Table S2** Measurement of perceived integration

| Questions (strongly disagree = 1, disagree = 2, neutral = 3, agree = 4, strongly agree = 5) | Mean | SD | Min | Max |
| --- | --- | --- | --- | --- |
| I have a strong sense of belonging to my community. | 3.24 | 0.70 | 1.00 | 5.00 |
| I have a strong emotional connection with my neighbors. | 2.98 | 0.66 | 1.00 | 5.00 |
| I trust my neighbors, who are locals. | 3.42 | 0.62 | 1.00 | 5.00 |
| I would like to be friends with locals. | 3.94 | 0.66 | 2.00 | 5.00 |
| I feel that locals do not like me (recode). | 3.91 | 0.72 | 1.00 | 5.00 |
| I feel that locals discriminate against me (recode). | 3.91 | 0.75 | 1.00 | 5.00 |
| *Average score (Cronbach’s* $\alpha$*=0.73)* | 3.57 | 0.45 | 1.33 | 4.67 |

**Table S3** Multilevel Generalized Structural Equation Modeling (MGSEM) results.

|  | Social interaction | | Perceived integration | | Mental health | |
| --- | --- | --- | --- | --- | --- | --- |
|  | *β* | *S.E.* | *β* | *S.E.* | *β* | *S.E.* |
| **Public spaces** |  |  |  |  |  |  |
| Typical public spaces |  |  |  |  |  |  |
| Sidewalks | 0.077 | 0.054 | 0.114^**^ | 0.047 | 0.051 | 0.059 |
| Parks | 0.026 | 0.049 | -0.012 | 0.041 | 0.241^***^ | 0.055 |
| Public transport stations | -0.129^*^ | 0.069 | -0.145^**^ | 0.060 | -0.263^***^ | 0.074 |
| Semi-public spaces |  |  |  |  |  |  |
| Nightlife spots | -0.019 | 0.058 | -0.029 | 0.051 | 0.000 | 0.062 |
| Restaurants | 0.033 | 0.076 | 0.044 | 0.065 | 0.193^**^ | 0.082 |
| Privately owned public spaces |  |  |  |  |  |  |
| Physical-spatial quality | -0.027 | 0.047 | 0.092^**^ | 0.040 | 0.111^**^ | 0.051 |
| Social-spatial quality | -0.124^***^ | 0.046 | -0.015 | 0.040 | 0.102^**^ | 0.051 |
| **Mediators** |  |  |  |  |  |  |
| Social interaction |  |  | 0.258^***^ | 0.036 | -0.051 | 0.037 |
| Perceived integration |  |  |  |  | 0.160^***^ | 0.038 |
| **Control variables** |  |  |  |  |  |  |
| Age (*ref*: 18–40) | 0.233^***^ | 0.088 | -0.101 | 0.083 | 0.141^*^ | 0.085 |
| Gender (*ref*: Male) | -0.130^*^ | 0.077 | -0.128^*^ | 0.073 | 0.106 | 0.073 |
| Education (*ref*: High school or below) | -0.125 | 0.086 | 0.154^*^ | 0.081 | 0.146^*^ | 0.084 |
| Personal annual income (*ref*: <50,000 CNY) | 0.097 | 0.081 | 0.365^***^ | 0.076 | 0.226^***^ | 0.078 |
| Constant | 0.042 | 0.095 | -0.186^**^ | 0.088 | -0.340^***^ | 0.095 |

Note: * *P* < 0.10, ** *P* < 0.05, *** *P* < 0.01. All continuous variables are standardized.
